# The characteristics of chest HRCT and pulmonary function tests in lung‐onset primary sjogren's syndrome

**DOI:** 10.1002/iid3.957

**Published:** 2023-08-08

**Authors:** Xin Dong, Yanli Gao, Man Li, Dong Wang, Jifeng Li, Yongfeng Zhang

**Affiliations:** ^1^ Department of Rheumatology, Beijing Chaoyang Hospital Capital Medical University Beijing China; ^2^ Department of Radiology, Beijing Chaoyang Hospital Capital Medical University Beijing China; ^3^ Department of Respiratory and Critical Care Medicine, Beijing Institute of Respiratory Medicine and Beijing Chao‐Yang Hospital Capital Medical University Beijing China

**Keywords:** chest high‐resolution computerized tomography, interstitial lung disease, pulmonary function tests, Sjögren's syndrome

## Abstract

**Introduction:**

Interstitial lung disease (ILD) can manifest before the diagnosis of primary Sjögren's syndrome (pSS), however, the underlying mechanisms remain unclear. The aim of this study is to investigate the characteristics of lung‐onset pSS using chest high‐resolution computerized tomography (HRCT) and pulmonary function tests (PFTs).

**Methods:**

The data of 102 patients with pSS‐ILD were retrospectively analyzed. The patients were divided into two groups: lung‐onset group and the nonlung‐onset group. The chest HRCT, PFTs, and clinical and laboratory data were evaluated and compared.

**Results:**

Among the 102 patients with pSS‐ILD, 59 (57.8%) were lung‐onset and 43 (42.2%) were nonlung‐onset. Chest HRCT in the lung‐onset group showed higher percentage of usual interstitial pneumonia and nonspecific interstitial pneumonia, the difference did not reach statistical significance. The total HRCT score was higher in the lung‐onset group, compared with the nonlung‐onset group (2 [2, 3], vs. 2 [1, 2], *p* = .014). Total lung capacity (TLC) (%pred) [(75.4 ± 16.2) versus (82.8 ± 19.4), *p* = .049] and forced vital capacity (FVC) (%pred) [(82.2 ± 19.9) versus (91.6 ± 28.3), *p* = .050] were significantly lower in the lung‐onset group, compared with the nonlung‐onset group. Residual volume (RV)/TLC (%) significantly increased more than 40% in the lung‐onset group (*p* = .015). Restricted ventilation disorder, small airway obstruction and reduced diffusing capacity of the lung for carbon monoxide/alveolar volume (%Pred) were more common in the lung‐onset group (*p* = .038, *p* = .050, and *p* = .050, respectively). Correlation analysis showed that HRCT score was positively correlated with the interval between the onset of pulmonary symptoms and the diagnosis of ILD, serum CA125, and serum CEA. TCL (%pred), VC (%pred), FVC (%pred) were negatively correlated with serum CA125.

**Conclusion:**

Lung‐onset is common in pSS patients with more severe lung function impairments. Serum biomarkers, such CA125, CEA, and ALB, were associated with the severity of lung damage.

## Graphical abstract

Lung‐onset is common in primary Sjögren's syndrome patients, and these patients present with more severe lung function impairments. Serum biomarkers, such CA125, CEA, and ALB were associated with the degree of lung damage.

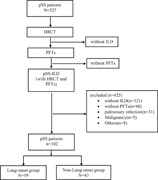

## 1 INTRODUCTION

Primary Sjogren's syndrome (pSS) is a progressive autoimmune disease with unique characteristics, including lymphocyte infiltration in the exocrine glands and loss of secretory function. As a systemic autoimmune exocrine disease, pSS affects multiple organs, including blood, gastrointestinal system, kidney, skin, lungs, and nervous system.^1^ Sicca symptom is one of the most important symptoms based on the classification criteria of pSS.^2^, ^3^ Nonsicca symptoms (or extra glandular manifestations [EGM]), especially pulmonary symptoms, can also be part of the initial manifestation in many pSS patients.

High‐resolution computed tomography (HRCT) scans have shown lung changes in 34%–50% of pSS patients.^4^ Lung changes can lead to poor quality of life and reduce the survival rate to 10 years.^5^ Interstitial lung disease (ILD) can manifest before the initial diagnosis of pSS.^6^ The interval between systemic manifestation and the pSS diagnosis is approximately 10 years.^7^

Our previous studies indicate that the proportion of nonsicca‐onset is higher in the pSS‐ILD patients. Functional impairments of the lungs are associated with poor survival rate.^8^ However, few studies have investigated the characteristics of pSS‐ILD patients with lung‐onset. In this study, we investigated the characteristics of chest radiological and pulmonary function tests (PFTs) of pSS‐ILD patients with lung‐onset.

## 2 MATERIALS AND METHODS

### 2.1 Study design and participants

**Figure 1:**
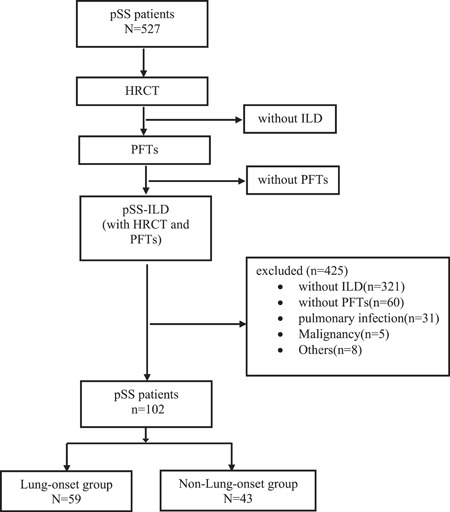
Flow chart of screening of the study participants. A total of 527 pSS patients who completed chest HRCT were screened. The following patients were excluded from the study: 321 patients without ILD, 60 patients without PFTs, 31 patients with pulmonary infection, five patients with malignancy, and eight patients with others complications. A total of 102 patients were included in this study and divided into two groups according to the onset: lung‐onset versus nonlung‐onset groups. HRCT, high‐resolution computerized tomography, ILD, interstitial lung disease, PFTs, pulmonary function tests, pSS, primary Sjogren's syndrome.

This was an observational and retrospective study. A total of 527 pSS patients were screened at Beijing Chaoyang Hospital between 2011 and 2020. One hundred and two patients with pSS‐ILD were included (Figure 1). The 102 patients were divided into two groups according to the disease onset: lung‐onset group and nonlung‐onset group.^9^ The clinical and imaging data of these two groups were analyzed.

Patients diagnosed with pSS based on 2002 International Classification Criteria for Sjögren Syndrome^2^ or 2016 ACR/EULAR classification criteria for Sjögren's syndrome, were recruited.^3^ The diagnosis of ILD was based on abnormal HRCT without infection, tumor or occupational exposure. The pSS with lung‐onset is defined as follows: (1) Chronic cough or exertional dyspnea as the initial manifestation; (2) abnormal HRCT in the presence of established ILD. Patients with lung damage unrelated to pSS, including asthma, chronic obstructive pulmonary disease, and other chronic lung diseases, were excluded from the analysis. The pSS with nonlung‐onset was defined as follows: (1) Initial manifestation met two criteria of international classification criteria for Sjögren Syndrome; (2) Without sicca symptoms, but having supplementary examination on classification criteria or suspicion of pSS from the EULAR pSS Disease Activity Index questionnaire (at least one domain). The exclusion criteria of pSS were based on ACR/EULAR (2016 criteria).

### 2.2 Data collection

Clinical data at disease onset were collected, including first manifestations, time, symptoms, chest HRCT and PFTs, demographics (gender and age at diagnosis), history of disease, smoking history, clinical symptoms (oral symptoms, ocular symptoms, swelling of parotid gland, arthralgia, Raynaud's phenomenon, fatigue, weight loss, cough, exertional dyspnea), physical signs, and laboratory examinations.

Laboratory examinations include the following: the levels of immunoglobulins (IgG, IgM, and IgA), serum complement C (C3 and C4), C‐reactive protein (CRP), Rheumatoid factor (RF) and erythrocyte sedimentation rate (ESR), lactate dehydrogenase (LDH), albumin (ALB), tumor markers (CA19‐9, CA125, CEA), as well as the presence of antinuclear antibodies (ANA), anti‐Ro/SSA and anti‐La/SSB.

### 2.3 Lung evaluation

The classification of chest HRCT was conducted by two experienced radiologists. HRCT patterns were categorized according to the 2013 international multidisciplinary classification of idiopathic interstitial pneumonia (IIP),^10^ as usual interstitial pneumonia (UIP), nonspecific interstitial pneumonia (NSIP), organizing pneumonia (OP), lymphocytic interstitial pneumonia (LIP), and “unclassified” subgroups. The radiological UIP criteria were applied as those for the diagnosis of idiopathic pulmonary fibrosis (IPF).^11^ NSIP was defined as the predominance of ground‐glass opacity (GGO), possible visible subpleural sparing and fine reticulation with minor or no honeycombing. OP was defined as single or multiple patchy consolidations. LIP was defined as multiple cysts and possible GGO.

The HRCT findings in each case of interstitial lung abnormalities (ILA) was scored by a four‐point scale (1%–25% involvement of 1; 26%–50% involvement of 2; 51%–75% involvement of 3;76%–100% involvement of 4). The predominant distribution of lesions was classified as being predominantly peripheral, peribronchovascular, or mixed.^12^

The following PFTs data were obtained from patient charts: total lung capacity (TLC), vital capacity (VC), forced expiratory volume in 1 s (FEV1), forced vital capacity (FVC), residual volume (RV), forced expiratory flow (FEF25/50/75) and diffusing capacity of the lung for carbon monoxide/alveolar volume (DLCO/VA). PFTs were shown as percentages of the predicted value of each parameter for each individual based on the age, gender and height. The damages to lung function mainly include obstructive ventilation disorder (OVD).

[The ratio of FEV1 to FVC: FEV1/FVC (%) <70%; and the ratio RV to TLC: RV/TLC (%) >35%], restricted ventilation disorder (RVD) [TLC (%Pred), VC (%Pred) and FVC (%Pred) <80%], small airway obstruction (SAO) [MEF25‐50 (%Pred) <70%, normal MEF75 (%Pred)], reduced diffusion capacity (RDC) [DLCO/VA (%Pred) <80%] and mixed ventilatory dysfunction (MVD) (OVD and RVD).^13^

### 2.4 Statistical analysis

SPSS 22.0 software was used for statistical analysis. Quantitative data was expressed as Mean ± standard deviation (SD). Continuous variables were expressed as either mean ± SD or Median and range. Independent samples *t* test or Mann–Whitney *U* test were used for two group comparisons. One‐way analysis of variance and LSD were used for multiple comparisons. Frequencies were analyzed using *χ* ^2^ test or Fisher's exact test. Pearson correlation coefficient was used for the correlation between two continuous variables. Results were considered statistically significant if *p* < .05.

## 3 RESULTS

### 3.1 Demographic characteristics and manifestations

Among the 102 pSS patients with ILD, 91 (89.2%) were female, 11 (10.8%) were male. The mean age of ILD‐onset, pulmonary symptoms‐onset and nonlung symptoms‐onset were (62 ± 10) years, (61 ± 10) years, and (58 ± 11) years, respectively. The median respiratory duration and non‐respiratory symptoms duration were 12 (2, 48) months and 36 (8, 84) months, respectively. Only 15 (14.7%) patients were smokers, and the duration of smoking was 20 (10, 20) years (Table 1).

**Table 1:** Clinical characteristics in pSS‐ILD patients with lung onset versus nonlung onset.

	Total	Lung onset group	Nonlung onset group	*p*
*Clinical data*				
*N* (%)	102	59 (57.8)	43 (42.2)	
Gender, female (%)	102 (89.2)	51 (86.4)	40 (93)	.290
ILD‐onset age, years	(62 ± 10)	(60 ± 10)	(64 ± 10)	.006*
Pulmonary symptoms‐onset age, year	(61 ± 10)	(58 ± 10)	(64 ± 10)	.009*
Nonlung‐onset age, year	(58 ± 11)	(58 ± 11)	(58 ± 12)	.959
Disease duration, months (median)	30 (6, 84)	12 (3, 60)	60 (24, 120)	.000*
Time of sicca to ILD, months (median)	24 (0, 63)	0 (0, 24)	60 (24, 120)	.000*
Time of pulmonary symptoms to ILD, months	6 (1.2, 24)	12 (3, 60)	2 (0, 12)	.000*
With ILD‐onset, *n* (%)	40	40 (67.8)	0	.000*
Somker, *n* (%)	15 (14.7)	10 (16.9)	5 (11.6)	.454
Dry mouth, *n* (%)	74 (72.5)	34 (57.6)	40 (93)	.000*
Dry eyes, *n* (%)	62 (60.8)	42 (47.5)	34 (79.1)	.001*
Parotid enlargement, *n* (%)	7 (6.9)	2 (3.4)	5 (11.6)	.140
Saprodontia	35 (34.3)	13 (22)	22 (51.2)	.002*
Minor salivary gland biopsy, *n* (%)	28 (27.5)	21 (35.6)	7 (16.3)	.031*
Arthritis, *n* (%)	16 (15.7)	7 (11.9)	9 (20.9)	.214
Raynaud's, *n* (%)	10 (9.8)	4 (6.8)	6 (14)	.229
Dry cough, *n* (%)	79 (77.5)	52 (88.1)	27 (62.8)	.002*
Panting after activity, *n* (%)	73 (71.6)	48 (81.4)	25 (58.1)	.010*
Crackles	68 (66.7)	45 (76.3)	23 (53.5)	.016*
*Laboratory data*				
ANA ≥ 1:100, *n* (%)	87 (85.3)	48 (81.4)	39 (90.7)	.118
Anti‐Ro/SSA52KD(+), *n* (%)	63 (61.8)	36 (61.0)	27 (62.8)	.856
Anti‐Ro/SSA60KD(+), *n* (%)	44 (43.1)	22 (37.3)	22 (51.2)	.162
Anti‐Ro/SSA(+), *n* (%)	76 (74.5)	43 (72.9)	33 (76.7)	.658
Anti‐La/SSB(+), *n* (%)	20 (19.6)	8 (13.6)	12 (27.9)	.072
RF(+), *n* (%)	30 (19.6)	14 (23.7)	16 (37.2)	.140
*Laboratory results*				
Serum IgG, mg/dL	1630 (1320, 2165)	1570 (1215, 1990)	1740 (1380, 2440)	.031*
IgA, mg/dL	314 (218, 417)	295 (200, 337)	353 (228, 497)	.039*
IgM, mg/dL	129 (188, 417)	125 (81, 178)	131 (99, 207)	.201
Serum C3, mg/dL	98 (87, 108)	99 (90, 108)	94 (81, 108)	.338
C4, mg/dL	19.5 (16.5, 23.4)	19 (16, 24)	20 (17, 22)	.425
CA19‐9, U/mL	9.2 (5.0, 21.5)	7.3 (4.3, 25.4)	11.1 (6.6, 20.0)	.382
CA125, U/mL	17.0 (12.0, 30.9)	18.3 (12.9, 32.0)	14.8 (10.0, 30.0)	.176
CEA, ng/mL	2.0 (1.1, 3.5)	2.1 (1.1, 3.8)	1.8 (1.1, 2.5)	.190
LDH, U/mL	200 (166, 242)	204 (180, 251)	182 (159, 227)	.018*
ALB, g/L	35.3 (31.4, 38.2)	34.9 (31.8, 38.2)	35.7 (30.8, 38.3)	.786

*Note*: Data are presented as either mean ± SD or median (Q1, Q3).

Abbreviations: ALB, albumin; ANA, antinuclear antibody; C3, complement 3; C4, complement 4; CA125, cancer antigen 125; CA19‐9, cancer antigen 19‐9; CEA, Carcinoembryonic antigen; CRP, C‐reactive protein; ESR, erythrocyte sedimentation rate; F/M, Female to male ratio; IgA, immunoglobulins A; IgG, immunoglobulins G;  LDH, lactate dehydrogenase; *n* (%), the number of cases (percentage); RF, rheumatoid factor.

* *p* < .05.

The manifestation of lung‐onset patients were pulmonary symptoms (shortness of breath and/or coughing, *n* = 33, 32.4%) or ILD (*n* = 26, 25.5%). The most common manifestation at onset was sicca symptoms (dryness of the eyes and oral mucosa, *n* = 43, 42.2%), joint involvement (arthralgia and/or arthritis, *n* = 16, 15.7%), Raynaud's phenomenon (*n* = 10, 9.8%), saprodontia (*n* = 35, 34.3%), parotid gland swelling (*n* = 7, 6.9%), and minor salivary gland biopsy positive (*n* = 28, 27.5%).

Median diagnosis time (nonlung‐onset group) from pSS‐related manifestations to pSS‐ILD diagnosis was 24 (0, 63) months. Only 4 (3.9%) patients had ILD diagnosis before pSS with the median diagnosis time of 41 (9.3, 60) months. The median diagnosis time from pulmonary‐onset to pSS‐ILD was 6 (1.2, 64) months. Although the presence of sicca symptoms was the most common manifestation, 26 (25.5%) patients were diagnosed with pSS based on lung manifestation.

Autoantibody analyses revealed that anti‐Ro/SSA and anti‐La/SSB were positive in 74.5% (76/102) and 19.6% (20/102) of pSS‐ILD patients. A total of 28 (27.5%) patients underwent a labial salivary gland (LSG) biopsy. Percentages of patients with ANA and RF were 85.3% (87/102) and 29.4% (30/102), respectively.

### 3.2 Lung HRCT and PFTs features

HRCT patterns of 102 patients were analyzed. The most frequent HRCT finding was the multiple HRCT patterns (34.3%) coexisted with OP, NSIP, and UIP, followed by the single HRCT pattern (51%), such as UIP, NSIP, Unclassified pattern, OP, and LIP (Table 2). The median HRCT score was 2 (2, 3). HRCT findings of the distribution of lesions and emphysema were evaluated. The main distribution of abnormalities was predominantly peripheral (33%), peribronchovascular (11%), and mixed (56%) (Table 3).

**Table 2:** HRCT patterns in pSS‐ILD patients with lung onset versus non‐lung onset.

	Total	Lung onset group	Nonlung onset group	*p*
HRCT pattern, *n* (%)	102	59 (57.8)	43 (42.2)	
Single HRCT pattern	52 (51)	32 (54.2)	20 (46.6)	
UIP (single)	21 (20.6)	14 (23.7)	7 (16.3)	.358
NSIP (single)	20 (19.6)	13 (22)	7 (16.3)	.470
OP (single)	8 (7.8)	4 (6.8)	4 (9.3)	.640
LIP	3 (2.9)	1 (1.7)	2 (4.7)	.383
Multiple(OP/NSIP/UIP)	35 (34.3)	19 (32.2)	16 (37.2)	.599
With NSIP feature	32 (91.4)	13 (22)	19 (44.2)	.017*
With OP feature	29 (82.9)	17 (28.8)	12 (27.9)	.920
Unclassified	16 (15.7)	9 (15.3)	7 (16.3)	.888

Abbreviations: LIP, Lymphocytic interstitial pneumonia); n (%), the number of cases (percentage); NSIP, nonspecific interstitial pneumonia; OP, organizing pneumonia; UIP, usual interstitial pneumonia.

* *p* < .05.

**Table 3:** The lesion distribution of ILA in pSS‐ILD patients with lung onset versus non‐lung onset.

	HRCT findings, *n* (%)	Total	Lung onset group	Nonlung onset group	*p*
	*N*	100	58	42	
Distribution	Predominantly peripheral, *n* (%)	33 (33)	18 (31)	15 (35.7)	.560
	Peribronchovascular, *n* (%)	11 (11)	8 (13.8)	3 (7.1)	
	Mixed, *n* (%)	56 (56)	32 (55.2)	24 (57.1)	
	*N*	76	44	32	
Location	Upper lobes, *n* (%)	9 (9)	3 (5.2)	6 (14.3)	.271
	Lower lobes, *n* (%)	16 (16)	9 (15.5)	7 (16.7)	
	Diffuse, *n* (%)	75 (75)	46 (79.3)	29 (69)	
Emphysema		9 (11.8)	5 (11.4)	4 (12.5)	.880

Abbreviation: *n* (%), the number of cases (percentage).

PFTs findings indicate that TLC (%Pred) (78.61 ± 18.69) was less than 80%, VC (%Pred) (85.45 ± 23.07) and FVC (%Pred) (86.21 ± 24.19) were more than 80%; FEV1 (%Pred) (82.34 ± 21.97), FEV1/FVC (%) (80.50 ± 8.29) were more than 80%; and RV/TLC (%) (41.66 ± 8.14) was more than 35%, MEF25 (%Pred) (61.48 ± 29.38) and MEF50 (%Pred) (67.34 ± 27.37) were less than 70%, MEF75 (%Pred) (83.69 ± 35.11) was more than 80%; DLCO/VA (%Pred) (68.08 ± 24.95) was less than 70% (Table 4). RDC was 92.1%, followed by SAO (48%), RVD (39.2%), OVD (17.6%), and MVD (3.9%) (Table 4).

**Table 4:** PFTs in pSS‐ILD patients with lung onset versus nonlung onset.

PFT index	Total	Lung onset group	Nonlung onset group	*p*
TCL, (%Pred)	78.61 ± 18.69	75.4 ± 16.2	82.8 ± 19.4	.049*
VC, (%Pred)	85.45 ± 23.07	81.9 ± 19.4	90.2 ± 26.7	.074
FVC, (%Pred)	86.21 ± 24.19	82.2 ± 19.9	91.6 ± 28.3	.050
FEV1, (%Pred)	82.34 ± 21.97	78.8 ± 17.6	87.1 ± 26.3	.062
FEV1/FVC (%)	80.50 ± 8.29	81.0 ± 7.1	79.9 ± 9.7	.512
RV/TLC (%)	41.66 ± 8.14	39.9 ± 8.7	43.9 ± 6.8	.015*
MEF25, (%Pred)	61.48 ± 29.38	62.9 ± 31.4	59.6 ± 26.8	.584
MEF50, (%Pred)	67.34 ± 27.37	65.8 ± 24.9	69.4 ± 30.5	.523
MEF75, (%Pred)	83.69 ± 35.11	82.6 ± 35.7	85.0 ± 34.7	.738
DLCO/VA, (%Pred)	68.08 ± 24.95	64.7 ± 24.9	72.7 ± 24.6	.113
RVD, *n* (%)	40 (39.2)	28 (48.3)	12 (27.9)	.038*
OVD, *n* (%)	18 (17.6)	10 (17.2)	8 (18.6)	.859
SAO, *n* (%)	49 (48)	33 (56.9)	16 (37.2)	.050
MVD, *n* (%)	4 (3.9)	1 (1.7)	3 (7)	.309
RDC, *n* (%)	93 (92.1)	56 (96.6)	37 (86)	.050

Abbreviations: (%Pred), the percentage of the predicted value of each parameter; FEV1, forced expiratory volume in 1 s; FVC, forced vital capacity; MEF25, 25% forced expiratory flow rate; MEF50, 50% forced expiratory flow rate; MEF75, 75% forced expiratory flow rate; MVD, mixed ventilatory dysfunction; *n* (%), the number of cases (percentage); OVD, obstructive ventilatory dysfunction; PFT, pulmonary function test; RDC, reduced diffusion capacity; RVD, restricted ventilation disorder; SAO, small airway obstruction; TCL, total lung capacity; VC, vital capacity.

* *p* < .05.

### 3.3 Comparisons of demographic, clinical characteristics and immunological data between the lung‐onset and nonlung‐onset groups

The clinical characteristics are shown in Table 1. Gender was well matched. Compared with the nonlung‐onset group, the age of ILD and pulmonary symptoms onset were significantly younger in the lung‐onset group (*p* = .006, *p* = .009, respectively). The interval between pSS‐related manifestation and ILD diagnosis was significantly longer in the non‐lung‐onset group (*p* = .000). The interval between pulmonary symptoms onset and ILD was significantly longer in the lung‐onset group (*p* = .000). In the lung‐onset group, 40 cases initiated with ILD, and four cases had ILD before pSS.

Ten (16.9%) patients were smokers in the lung‐onset group, and 5 (11.6%) patients were smokers in the nonlung‐onset group (*p* = .454). Sicca symptoms (dryness of oral mucosa and the eyes, saprodontia) were more common in the nonlung‐onset group (*p* = .000, *p* = .001, and *p* = .002, respectively). Pulmonary symptoms (dry cough, shortness of breath after activity, and crackles) were more predominant in the lung‐onset group (*p* = .002, *p* = .01, and *p* = .016, respectively).

The case–control study revealed that ANA, anti‐Ro/SSA, anti‐La/SSB, and RF presence were similar between two groups. The serum IgG and IgA titers were significantly higher in the nonlung‐onset group (*p* = .031 and *p* = .039, respectively), whereas IgM, C3 and C4 were similar. Serum LDH level was higher in the lung‐onset group (*p* = .018). The serum level of CEA and CA125 were similar between two groups (Table 1).

### 3.4 Comparisons of radiological characteristics and pulmonary functions between nonlung‐onset and lung‐onset pSS‐ILD patients

In nonlung‐onset group, multiple pattern (OP/NSIP/UIP) was most common, especially with NSIP feature (44.2% vs. 22%, *p* = .017). In lung‐onset group, the percentage of UIP and NSIP were higher, but the difference did not reach statistical significance (Table 2).

In addition, 59 patients in the lung‐onset group and 43 patients in the nonlung‐onset group underwent HRCT. The total HRCT score was higher in the lung‐onset group (2[2, 3] vs. 2[1, 2], *p* = .014).

HRCT findings of abnormalities, distribution and emphysema were compared between the lung‐onset and the nonlung‐onset groups. The distribution characteristics were similar between two groups (*p* = .560, *p* = .271, and *p* = .880) (Table 3). Three patients showed lung bullae, two in the nonlung‐onset group, and one in the lung‐onset group, respectively.

PFTs (Table 4) indicates that TLC (%pred) and VC (%pred) were significantly lower in the lung‐onset group (*p* = .049 and *p* = .050, respectively). Compared with the nonlung‐onset group, RV/TLC (%) significantly increased more than 40% in the lung‐onset group (*p* = .015). MEF25 (%Pred), MEF50 (%Pred) and MEF75 (%Pred) were normal. DLCO/VA (%Pred) in both groups were less than 80% (Table 4).

RVD and SAO were more common in the lung‐onset group (*p* = .038 and *p* = .050, respectively), whereas the percentages of OVD and MVD were similar (*p* = .859 and *p* = .309, respectively). RDC was predominant in both lung‐onset and nonlung‐onset groups (56[96.6%] vs. 37[86%]).

### 3.5 Correlation analysis

Correlation analysis showed that HRCT score was positively correlated with the interval between pulmonary symptoms onset and the diagnosis of ILD (months) (*p* = .027, *r* = .238), but was not correlated with the interval between the onset of pSS‐related manifestation and ILD diagnosis.

HRCT score was positively correlated with serum level of CA125 (*p* = .006, *r* = .285) and CEA (*p* = .017, *r* = .245). However, HRCT score was negatively correlated with serum ALB concentration (*p* = .034, *r* = ‐0.210). There was no correlation with other immune indexes. HRCT score was negatively correlated with TCL (%pred) (*p* = .002, *r* = ‐0.313), VC (%pred) (*p* = .003, *r* = ‐0.291), FVC (%pred) (*p* = .002, *r* = −.300), FEV1 (%pred) (*p* = .018, *r* = −.236) and DLCO/VA (%Pred) (*p* = .004, *r* = −.285). TCL (%pred) was negatively correlated with serum LDH concentration (*p* = .025, *r* = −.225). DLCO/VA (%Pred) was postively correlated with serum ALB concentration (*p* = .010, *r* = .255). TCL (%pred) (*p* = .015, *r* = ‐0.259), VC (%pred) (*p* = .030, *r* = −.231) and FVC (%pred) (*p* = .029, *r* = −.232) were negatively correlated with serum level of CA125.

## 4 DISCUSSION

pSS is a chronic inflammatory autoimmune disorder. ILD presents severe extra‐glandular manifestations with the prevalence of lung involvement varying from 9% to 75%.^14^ 10% to 51% of patients have no sicca symptoms when ILD occurs, even if ILD could appear a few years earlier. In addition, some patients with ILD were seronegative, which causes challenges to the pSS diagnosis.^15^

The present study is the first one to investigate the features of chest HRCT and PFTs in the lung‐onset pSS patients. Our findings indicate that (1) the pulmonary symptoms‐onset age and ILD‐onset age were younger. (2) Chest HRCT features were more severe. (3) The small airway and alveolar impairment were severe. (4) Autoantibodies positive rate was lower. (5) HRCT score was positively correlated with elevated serum CA125 and CEA. (6) PFTs indexes [TCL (%pred), VC (%pred), FVC (%pred)] were negatively correlated with the elevated serum CA125.

The mean age of ILD‐onset [(61 ± 10) years] was similar to previous reports.^9^, ^16^ Here, we demonstrated that the pulmonary symptoms‐onset age and ILD‐onset age were older in the nonlung‐onset group, suggesting that pSS is a risk factor of ILD. The pulmonary symptoms‐onset age and ILD‐onset age were younger (*p* = .006 and *p* = .009, respectively) in the lung‐onset group. However, Gao et al. showed that the age between sicca‐onset and nonsicca‐onset group was similar.^9^ Whereas, Manfredi et al. reported pSS patients with ILD as the first clinical manifestation were older.^17^ Recently, studies of interstitial pneumonia with autoimmune features (IPAF) showed IPAF and connective tissue disease (CTD)‐ILD patients were significantly younger, with a higher proportion of female patients. A diagnosis of IPAF might predict a favorable prognosis.^18^ Therefore, younger female patients with lung symptoms‐onset should get screen CTD and receive early treatments.

In the present study, although sicca symptoms were the most common manifestation at the onset of pSS (42.2% of pSS‐ILD patients had oral mucosa and dryness of the eyes), more than half of the pSS‐ILD patients had pulmonary symptoms as the first manifestation, including dry cough (88.1%) and panting after activity (81.4%). In pSS patients, the most common symptom of airway diseases was cough with an incidence of 41%–61%. The severity of cough was correlated with the degree of airway inflammatory and poor quality of life.^6^ Studies have shown that there are correlations between the dysfunction of the tracheobronchial mucociliary clearance with the inflammatory lymphocytic infiltration and the dysfunction of mucosae impair secretion clearance. Conversely, the airway inflammation leads to bronchial hyperreactivity and results in bronchioles, pulmonary alveoli, and pulmonary parenchyma.^19^

In clinical practice, subclinical lung disease and ILD are common. In the lung‐onset group, 32.4% of patients started with pulmonary symptoms, 25.5% of patients were diagnosed with pSS due to ILD. Four cases had ILD before pSS. A Spanish multicenter study has reported 25 pSS patients with ILD. Among them, 60% had ILD before pSS.^20^ A study conducted by Gao et al. has shown that nearly half of the pSS‐ILD patients did not report sicca symptoms, which led to a significant delay in pSS diagnosis.^9^ Manfredi et al. have studied 77 pSS‐ILD patients without onset of sicca, however, 13 patients had lung damages.^17^ Therefore, sicca symptoms can be often overlooked, resulting in delayed diagnosis of pSS. Meanwhile, ILD in pSS is heterogeneous and insidious.^21^ Hence, the early detection of pSS could be a real challenge.

In the nonlung‐onset group, the median interval between the pSS‐related manifestation and ILD diagnosis was longer, whereas the median interval between pulmonary onset and ILD was shorter (2 months). Previous studies have indicated that pulmonary involvement presents in pSS patients with longer disease duration.^22^ Furthermore, these results suggest that pSS patients with respiratory symptoms onset can progress to ILD in a short time. Another report has indicated that among 105 pSS patients, 10% had respiratory symptoms at the time of diagnosis or within the first year of diagnosis. The prevalence increases to 20% (±4%) in the fifth year.^23^ In our cohort study, correlation analysis showed that HRCT score was positively correlated with the interval between pulmonary onset and ILD (months) (*p* = .027, *r* = .238). Therefore, patients should be screened for ILD after the onset of respiratory symptoms.

For pSS, ILD is traditionally associated with smoking, aging, hypergammaglobulinemia, increased RF or ANA titers, anti‐Ro/SSA or anti‐La/SSB positivity, elevated CRP, and reduced serum C3 levels.^24^ In our study, ANA, anti‐Ro/SSA, anti‐La/SSB, and RF were positive in 85.3%, 74.5%, 19.6%, and 19.6% of the patients, respectively. Alhamad et al. reported 50% of pSS‑ILD patients were negative for ANA, RF, anti‐Ro/SSA, and anti‐La/SSB.^16^ Therefore, in ILD patients, the diagnosis of pSS should not solely rely on serology.

Interestingly, in our study, the positive rate of antibodies and the serum IgG/IgA were lower in the lung‐onset group. Similarly, Gao et al. demonstrated a low anti‐Ro/SSA and anti‐La/SSB rate in the nonsicca group.^9^ Other studies have also shown that hypergammaglobulinemia, anti‐Ro/SSA and anti‐La/SSB were rarely found in ILD‐onset pSS without sicca syndrome.^17^, ^25^ These results indicate that there may be different immune pathogenesis between the lung‐onset and nonlung‐onset groups.

B and T cells play important roles in the pathogenesis of pSS. Previous studies have shown that the frequency of abnormal ocular and oral diagnostic tests were higher in anti‐La/SSB positive patients.^26^ Saliva from pSS patients contains anti‐Ro/SSA and anti‐La/SSB antibodies.^27^ The increased IgG and autoantibodies levels can be due to a significant increase in the number of memory B cells and plasma cells secreting IgG in the exocrine glands. Moreover, studies showed the levels of B cell activating factor (BAFF) and B cell prosurvival factor increased in serum and salivary gland in pSS patients,^28^ suggesting that exocrine gland damages are frequently accompanied with activation of B lymphocyte.

On the contrary, many bronchoalveolar lavage fluid (BALF) studies have indicated CD4+T lymphocytic alveolitis in pSS patients without respiratory symptoms.^21^, ^29^ In addition, without any symptomatic and radiographic abnormality, there are still many infiltrating CD4+T lymphocytes in bronchial and bronchiolar submucosa.^30^, ^31^, ^32^ Therefore, the different manifestations of pSS may be due to the different pathological mechanism of respiratory damage and exocrine glands involvement.

The most common HRCT type of ILD in pSS is NSIP (41%–45%), followed by UIP (10%), OP (4%) and LIP (4%–8%). Our study indicated that the most frequent HRCT finding was the multiple HRCT patterns coexisted with OP, NSIP and UIP, followed by UIP, NSIP, unclassified pattern, OP and LIP. Our previous study has demonstrated that 40% of patients are classified as coexistence of multiple HRCT modes, among which the mixed mode of NSIP and OP (43.9%) and the mixed mode of NSIP and UIP (35.4%) are more common.^33^ Other studies have also demonstrated that the combination of different imaging modes is common.^25^, ^34^, ^35^

Our study indicates that the NSIP feature is most common in the nonlung‐onset group. Gao et al. have demonstrated that NSIP pattern is the most common pattern in the sicca‐onset group, followed by UIP. There was no difference between nonsicca‐onset and sicca‐onset. Nevertheless, the proportion of LIP pattern was higher in the sicca‐group.^9^ Manfredi et al. showed 35.6% of patients with lung‐dominant pSS patients presented UIP pattern.^17^

The imaging features of pSS‐ILD are complex and may overlap with other diseases. There are combinations of multiple variables, such as ground glass opacity, consolidation, reticular abnormality, honeycomb, cyst, and so on. First, different features may affect a variety of anatomical structures, such as the airways, alveoli, blood vessels, pleura, and so on. Second, the coexistence of multiple variables, such as the mixture of NSIP and OP, the mixture of NSIP and UIP, and the mixture of NSIP and LIP, suggests that nonuniformity and overlap of histopathological abnormalities can coexist in one patient. The diversity may attribute to different immunological mechanisms or different stages of immunological injury in the lung. The prognostic indicators related to variable abnormalities of pSS‐ILD will be investigated in our future studies. In the present study, the lung‐onset group had a higher total HRCT score (*p* = .014), which is consistent with others studies.^9^ HRCT score is an independent risk factor of mortality, which reflects the severity of lung involvement in association with prognosis in patients with pSS.^36^

PFT in pSS can be restrictive and obstructive with low DLCO and small airway disease even in patients without pulmonary symptoms.^36^ Airway and pulmonary parenchyma diseases are usually found in pSS patients. The patients can have obstructive and restrictive pulmonary function patterns at the same time. Pulmonary parenchymal disease in patients with pSS is very likely to deteriorate over time.^37^ Study have shown that ventilation dysfunction and restrictive functional defects are more common in the nonsicca group, accompanied with low TLC (%Pred) and FVC (%Pred) value.^9^ Manfredi et al. showed that TLC (%Pred), FVC (%Pred), VC (%Pred) and DLCO/VA (%Pred) were similar to sicca‐onset and ILD‐onset.^17^ In this study, RVD, SAO and RDC were more common in the lung‐onset group (*p* = .038, *p* = .050, *p* = .050), accompanied with lower TLC (%Pred), VC (%Pred) and FVC (%Pred) values (*p* = .049, *p* = .074, *p* = .050). DLCO/VA (%Pred) and MEF25‐50 (%Pred) was less than 70% in the lung‐onset group. The PFT defects suggest that small airway and alveolar impairment are more prominent in the lung‐onset group. Correlation analysis shows that HRCT score was negatively correlated with TCL (%pred), VC (%pred), FVC (%pred), FEV1 (%pred), and DLCO/VA (%Pred).

Reduced DLCO, FVC impairment, and fibrosis are commonly seen in patients with ILD.^38^ Previous studies have also reported FEV1 (<60 %Pred), FVC(<60 %Pred), PEF(<60 %Pred), and shorter median overall survival.^36^ In this study, reduced DLCO/VA with FVC impairment was more prominent in the lung‐onset group, while FEV1 (%Pred) and FVC (%Pred) were lower in the lung‐onset group.

Our results indicate that patients with severe lung impairments are more likely to be diagnosed with lung disease other than pSS. The delayed diagnosis of pSS is often due to the nonspecificity of the initial symptoms.

Serum LDH is a traditional biomarker for ILD,^39^ and LDH levels are positively correlated with CTD‐ILD activity or severity.^40^ In our study, serum LDH level was higher in the lung‐onset group (*p* = .018), and was negatively correlated with TCL (%pred). In addition, hypoproteinemia was a risk factor for the progression of ILD and an independent risk factor for mortality in pSS‐ILD patients.^8^, ^41^ In our cohort, the serum level of ALB significantly decreased in both groups. HRCT score was negatively correlated with serum ALB. Moreover, reduced DLCO/VA was correlated with hypoproteinemia. ALB decreases the production of oxygen free radicals, presents endothelial cell apoptosis, and inhibits platelet aggregation.^42^ The increased inflammatory factors in ILD can decrease the ALB synthesis in hepatocytes. Reduced serum ALB may increase active fibroblasts and aggravate pulmonary fibrosis. Therefore, ILD patients with hypoalbuminemia should receive more attention during diagnosis and treatment.

In recent years, tumor markers have been found to be associated with ILD in CTD. In this study, the serum level of CA125 and CEA were high in the lung‐onset group. However, HRCT score was positively correlated with serum CA125 and CEA. Moreover, elevated CA125 level was correlated with decreased TCL (%pred), VC (%pred) and FVC (%pred). A retrospective analysis of 706 patients with pSS showed that the serum level of CEA and CA125 were significantly elevated in pSS‐ILD patients, and CEA was significantly related to the onset of pSS‐ILD.^43^ Wang et al found that serum CEA concentration is negatively correlated with DLCO, and CEA can be used as an indicator for patients developing rapid progress ILD.^44^ The main etiopathogenesis of ILD is coexisting of continuous injury, the overactive repair and epithelial apoptosis with the development of fibroblast foci. The abnormal epithelial cells synthesize and release tumor markers. Therefore, the serum level of CA125 and CEA can reflect the function of epithelial cells, which can provide a new perspective to further explore the correlation between tumor markers and the severity of ILD.

The present study has several limitations. First, this is a retrospective study. Second, this was a single center study, which may limit the generalization of the results. Third, we only focus on the HRCT and PFTs of the pSS‐ILD patients, and the data on treatment and prognosis were not included. In the future, we will explore treatment and prognosis.

## 5 CONCLUSION

In conclusion, the present study is the first one that investigated the features of chest HRCT and PFTs in the lung‐onset pSS patients. Lung‐onset is common in pSS patients, which poses a challenge for rheumatologists to establish the diagnosis because of the lack of sicca symptoms and the lower presence of antiautoantibodies. Some respiratory symptoms can be the initial manifestation of pSS. High HRCT score, reduced DLCO/VA and FVC impairment are more common in the lung‐onset group. Serum biomarkers, such CA125, CEA and ALB are associated with the degree of lung damage. Therefore, pSS‐ILD patients with lung‐onset should receive effective treatments for their pulmonary damages.

## AUTHOR CONTRIBUTIONS

**Xin Dong**: Data curation; formal analysis; methodology; supervision; writing—original draft; writing—review and editing. **Yanli Gao**: Conceptualization; data curation; methodology; supervision. **Man Li**: Data curation. **Dong Wang**: Data curation; methodology. **Jifeng Li**: Data curation; formal analysis. **Yongfeng Zhang**: Conceptualization; data curation; formal analysis; methodology; software; validation; writing—original draft; writing—review and editing.

## CONFLICT OF INTEREST STATEMENT

The authors declare no conflicts of interest.

## Data Availability

I confirm that my article contains a Data Availability Statement even if no data is available (list of sample statements) unless my article type does not require one (e.g., Editorials, Corrections, Book Reviews, etc.). I confirm that I have included a citation for available data in my references section, unless my article type is exempt.
